# Noncontact atomic force microscopy study of the spinel MgAl_2_O_4_(111) surface

**DOI:** 10.3762/bjnano.3.21

**Published:** 2012-03-06

**Authors:** Morten K Rasmussen, Kristoffer Meinander, Flemming Besenbacher, Jeppe V Lauritsen

**Affiliations:** 1 Interdisciplinary Nanoscience Center and Department of Physics and Astronomy, Aarhus University, Ny Munkegade, DK-8000 Aarhus C, Denmark

**Keywords:** aluminium oxide, metal oxide surfaces, noncontact atomic force microscopy (NC-AFM), polar surfaces, reconstructions, spinel

## Abstract

Based on high-resolution noncontact atomic force microscopy (NC-AFM) experiments we reveal a detailed structural model of the polar (111) surface of the insulating ternary metal oxide, MgAl_2_O_4_ (spinel). NC-AFM images reveal a 6√3×6√3R30° superstructure on the surface consisting of patches with the original oxygen-terminated MgAl_2_O_4_(111) surface interrupted by oxygen-deficient areas. These observations are in accordance with previous theoretical studies, which predict that the polarity of the surface can be compensated by removal of a certain fraction of oxygen atoms. However, instead of isolated O vacancies, it is observed that O is removed in a distinct pattern of line vacancies reflected by the underlying lattice structure. Consequently, by the creation of triangular patches in a 6√3×6√3R30° superstructure, the polar-stabilization requirements are met.

## Introduction

While the application of metal oxides in, e.g., catalysis, gas sensors, fuel cells, high-*k* dielectrics and corrosion protection has seen a very strong development, fundamental research on the surface properties of metal oxides has been a topic of growing interest [1]. However, in many interesting cases the metal oxide is electrically nonconducting, which severely complicates the use of almost all traditional surface-sensitive techniques relying on the scattering or emission of charged particles. As a consequence the basic surface characterization and in particular a direct atomic-scale characterization of the surface structure is largely missing for a range of important metal oxides. In recent years, the noncontact atomic force microscope (NC-AFM) has been established as a unique tool to provide atomic-resolution real-space images of all types of flat surfaces regardless of the conductivity of the material, including many of the important insulating metal oxides [2–4]. The NC-AFM, applied to metal-oxide single-crystal surfaces under ultrahigh vacuum, thus allows the first detailed characterization of surface morphology down to the atomic scale of this important group of materials. In this work, a new surface-structure model of the MgAl_2_O_4_(111) surface, based on experimental NC-AFM data obtained on this surface, prepared under well-controlled ultrahigh vacuum (UHV) conditions, is presented.

**Figure 1:**
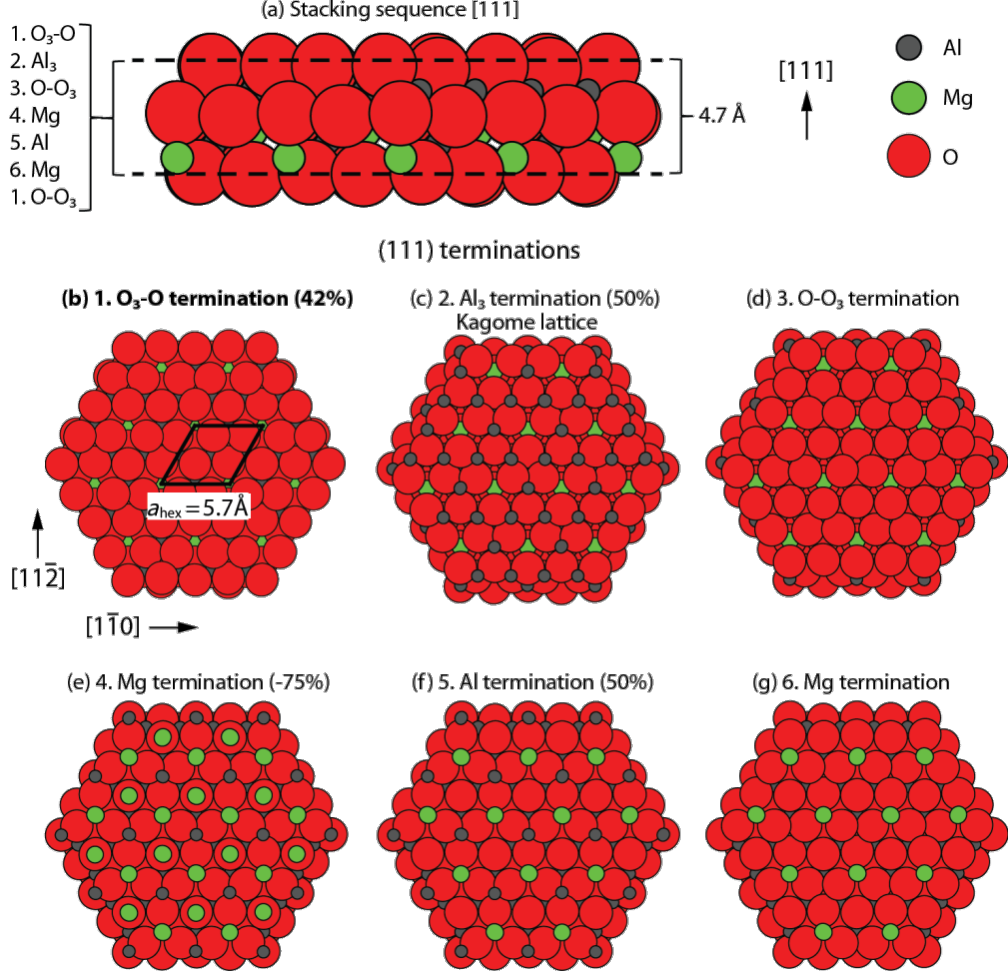
(a) Ball model of the MgAl_2_O_4_ stacking sequence in the [111] direction showing one repeat unit of 4.67 Å in height. (b–g) The hexagonal patches show six ball models, which illustrate the possible surfaces obtained from a bulk-truncation of the sequence in (a).

MgAl_2_O_4_ is a prototypical material with the so-called *spinel* structure, which defines a larger group of ionic materials with the AB_2_X_4_ stoichiometry [5]. For spinel, the repeat units perpendicular to any low-index surface normal consist of layers with alternating charge (see e.g., Figure 1a), and the surface terminations are therefore nominally polar and unstable in the truncated-bulk form [6–8]. The mechanisms that have been observed to lead to compensation of the surface dipole for such surfaces may strongly modify the surface relative to the truncated-bulk situation and are often divided into three groups: Change of the surface stoichiometry (reconstruction, terracing, etc.), adsorption of charge-compensating species from the residual gas (e.g., hydrogen), and electron redistribution between the top and bottom crystal faces. Depending on the material and the conditions under which the surface is kept, one or more of these stabilization mechanisms may be active, as previously observed, e.g., for ZnO [8–11]. It was recently shown that stabilization of the spinel MgAl_2_O_4_(100) surface may be achieved by a combination of cation redistribution in the surface layers and adsorption of hydrogen [12]. LEED experiments on thin Co_3_O_4_(111) films with the spinel structure [13–14] show evidence for an apparently unreconstructed (1×1) surface, the stability of which was proposed to be based on a Co^2+^/Co^3+^ inversion process leading to charge compensation. In the present case of MgAl_2_O_4_(111), no previous experimental studies are available concerning the surface structure. According to a theoretical study by Harding et al., and other theoretical studies [15–17], the oxygen-terminated MgAl_2_O_4_(111) is evaluated to be in its lowest energy state with 42% of the oxygen atoms removed from the oxygen crystal plane to fulfill the stabilization requirements. Furthermore, calculations show that dissociative adsorption of water up to a 90% coverage is very favorable and leads to high stability [18–19]. The NC-AFM data presented in this work reveal the MgAl_2_O_4_(111) surface to have a characteristic surface morphology consisting of triangular patches, the orientation and coverage of which are in agreement with the theoretical predictions for an oxygen-terminated surface with a certain percentage of the surface-layer atoms removed.

## Results and Discussion

**Figure 2:**
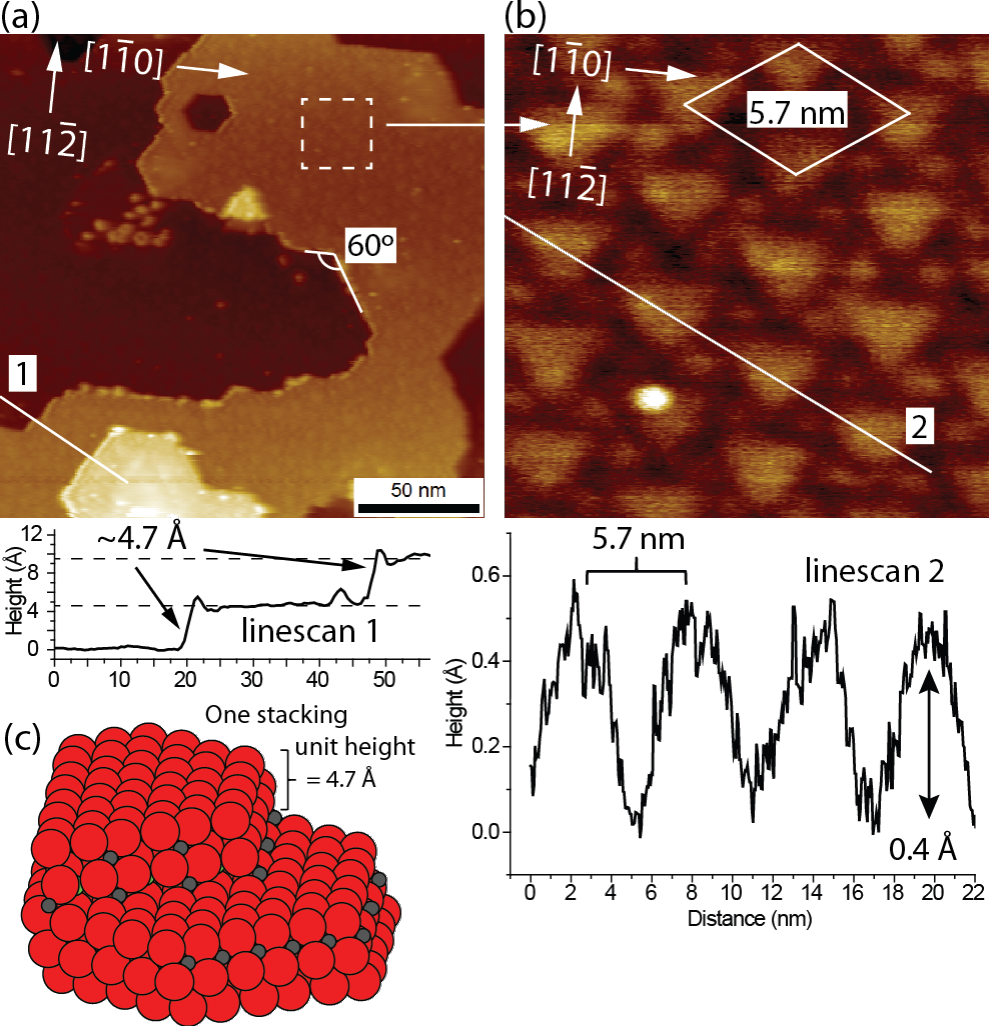
Experimental NC-AFM images recorded on the MgAl_2_O_4_(111) surface prepared by sputtering and annealing in oxygen (1150 °C, p(O_2_) = 1 × 10^−7^ mbar). (a) Large-scale NC-AFM image (200 × 200 nm^2^) of the surface (∆*f*_set_ = −15 Hz, *U*_bias_ = −8.5 V, *A*_p–p_ = 10 nm) showing step edges forming 60° angles. The graph for line scan 1 reflects the corrugation of a step. The position of the line scan is indicated in the image. (b) High-resolution zoom-in on a terrace (20 × 20 nm^2^), which shows a hexagonally ordered superstructure with a lattice parameter of 5.7 nm. The graph for line scan 2 shows the corrugation associated with the superstructure. The position of the line scan is indicated in the image. (c) Ball model of a single step edge on MgAl_2_O_4_(111) terminated by Al atoms.

Figure 2a shows a large-scale NC-AFM image of the freshly prepared MgAl_2_O_4_(111) sample revealing an almost perfectly flat surface. A few step edges are resolved on the surface, forming 60° angles, which reflects the hexagonal symmetry of surface atoms on the (111) surface. The bulk stacking sequence perpendicular to the surface in the [111] direction is somewhat complicated since it consists of 18 crystal layers in the form (O_3_O–Al_3_–OO_3_–Mg–Al–Mg)_3_. One of these three O_3_O–Al_3_–OO_3_–Mg–Al–Mg repeat units is illustrated in the side-view ball model in Figure 1a, indicating also the repeat-unit separation distance of a·**√**3/3 = 4.67 Å, where a = 8.08 Å [20]. Line scan 1 below Figure 2a shows the typical step-edge height measured at 4.7 ± 0.1 Å corresponding to the height of one O_3_O–Al_3_–OO_3_–Mg–Al–Mg repeat unit in the side-view ball model of Figure 1a. Because the measured step-edge height is always some multiple of 4.7 Å, it is assumed that the surface exposes only one type of termination. Figures 1b–g show top-view ball models of the six different possible (111) bulk-terminations obtained by truncating at each of the layers in the O_3_O–Al_3_–OO_3_–Mg–Al–Mg sequence. The six layers give rise to four types of surface termination: The surface can be terminated by either a hexagonally ordered oxygen plane (Figure 1b and Figure 1d), two different planes of Al^3+^ ions placed at octahedral sites (Figure 1c or Figure 1f), or a plane terminated by Mg^2+^ ions placed at tetrahedral sites (Figure 1e and Figure 1g). The distance between the O_3_ and O crystal planes is ~0.16 Å, which is why these planes in practice are considered as one single O surface layer [15]. As also indicated on the oxygen-terminated surface in Figure 1b, the primitive surface unit cell in the hexagonal representation, *a*_hex_, has a lattice constant of 5.72 Å. Considering that the crystal is prepared under an oxygen atmosphere we consider the surface terminated with an O_3_–O layer as the most probable candidate from these six models (Figure 1b), in particular since this is also predicted to be the overall most stable (111) termination [15–16]. To achieve a completely stable nonpolar surface of this kind it is furthermore calculated that 42% of the surface O anions in the O_3_ part of the O_3_–O layer should be removed [15]. It was previously suggested that formation of a corrugated surface would contribute to the overall stabilization of a polar surface. The effect is explained by the nonstoichiometry involved in the formation of step edges. In the case of Zn-terminated ZnO(0001), a stabilization mechanism was proposed involving the formation of preferentially O-terminated edges and pits, which effectively lowers the excess amount of Zn on this polar surface and reduces the surface dipole [21–22]. To evaluate the effect of step edges in the present case for MgAl_2_O_4_(111), a ball model is constructed in Figure 2c, which illustrates the structure of single step edges arising for an oxygen-terminated surface. When a step edge is created and terminated by aluminium cations (grey balls) in this model, more oxygen atoms (red balls) than Al atoms are removed from the surface, which could contribute to the surface stabilization according to the electrostatic criteria. However, the step-edge density in the large-scale NC-AFM images is seen to be far too low for this to be the primary stabilizing effect, and therefore other types of surface reconstructions must be present.

Higher-resolution NC-AFM images indeed reveal that the MgAl_2_O_4_(111) surface exposes a characteristic superstructure shown in the zoom image in Figure 2b (zoom-in area marked on Figure 2a with a dashed white square). The superstructure is observed to be composed of large triangular patches arranged in hexagonal symmetry described by a 5.7 nm unit cell, with each of the large triangles surrounded by six smaller protrusions, which also appear to have a triangular outline. For comparison, the rhombic unit-cell vector of the unreconstructed (1×1) surface is ten times smaller, i.e., *a*_hex_ = 5.72 Å (Figure 1b). Large-scale NC-AFM images furthermore show that the unit-cell vectors defining the superstructure are rotated by 30° as compared to the well-defined directions of the step edge (Figures 2a and 2b), i.e., the superstructure is most likely rotated by 30° compared to the basic unit-cell vectors of the (111) surface. Line scan 2 in Figure 2b shows that the apparent depth associated with the dark regions surrounding the triangles is measured to be approximately 0.4 Å in NC-AFM images. This corrugation is below the minimum distance of ~1.2 Å between two consecutive crystal planes in the [111] direction, i.e., between the O_3_–O and the Al_3_ layer (Figure 1a). However, it is well known that the atomic-level NC-AFM contrast in topographic measurements between two areas with a different chemical composition may be affected by work-function differences [23] or the structure and composition of the tip [2]. Furthermore, the size of the NC-AFM tip apex may hinder the accurate measurement of the true lowest point in the narrow geometrical depression between the triangles, and from this perspective it is reasonable to consider the dark region to reflect so-called line vacancies in which O atoms have been desorbed from the topmost O_3_–O layer.

**Figure 3:**
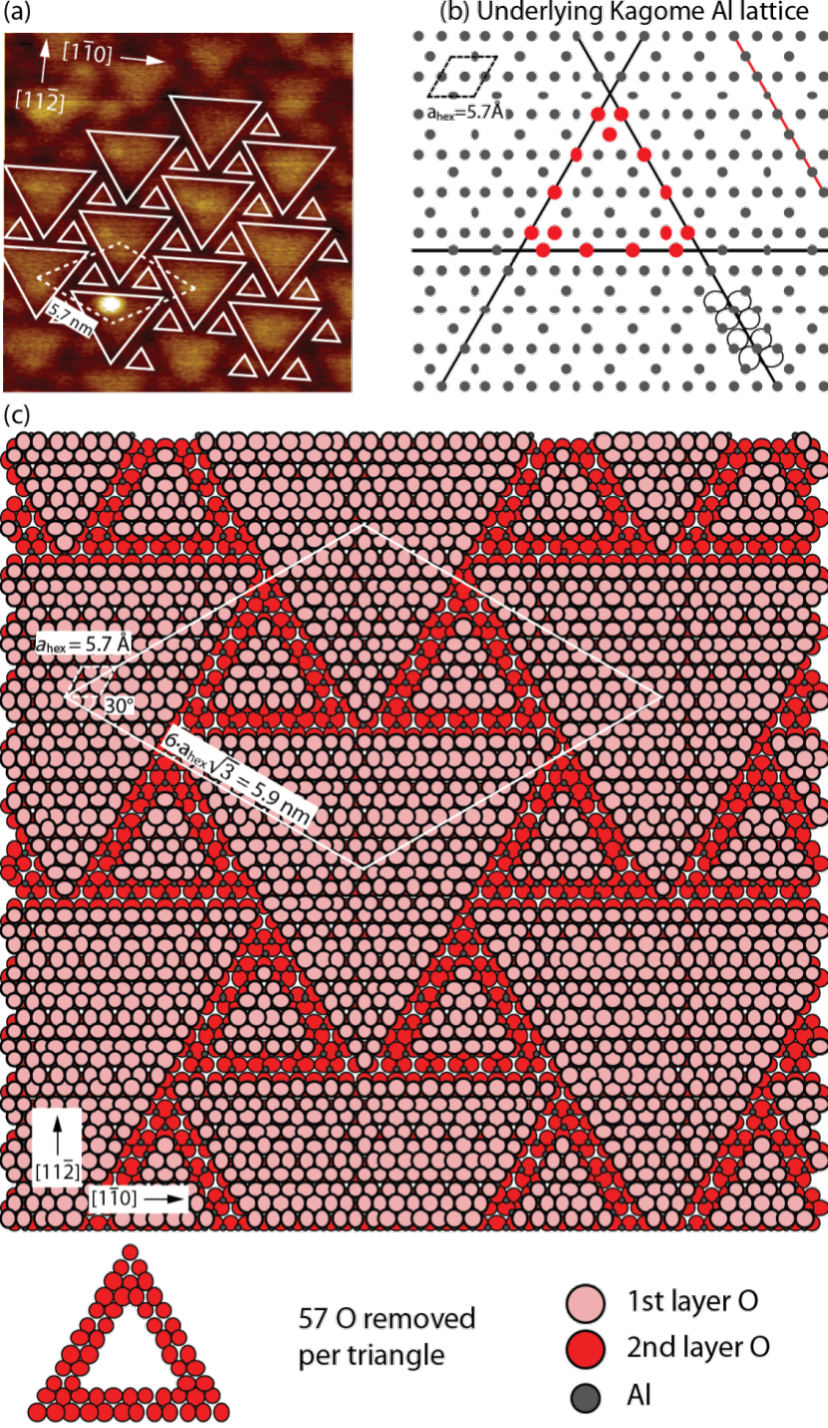
(a) Experimental NC-AFM image with the surface superstructure model superimposed. (b) Illustration of the subjacent aluminium lattice exposing a Kagomé pattern, which facilitates the formation of triangular patches. The O atoms are indicated along the black lines in order to illustrate the coordination along the lines. (Al: small grey, O: large, red). (c) Ball model illustrating the (6√3×6√3)R30° superstructure observed on the MgAl_2_O_4_(111) surface. The superstructure is created be the removal of oxygen atoms in triangular shapes.

Figure 3a shows a superstructure model superimposed on the NC-AFM image, which serves to illustrate the long-range hexagonal ordering of the two types of triangular patches. The experimentally observed superstructure, held together with the electrostatic stabilization criteria for the O_3_–O-terminated MgAl_2_O_4_(111), can now be utilized to construct a tentative structural model of the MgAl_2_O_4_(111) surface termination. The structural model has to comply with the symmetry and the dimensions obtained from the NC-AFM data, i.e., a superstructure with a unit cell approximately ten times the size, and rotated by 30°, relative to the primitive hexagonal surface unit cell with lattice parameter *a*_hex_ = 5.72 Å. Furthermore, the model has to expose two types of triangular patches, and the number of oxygen atoms has to be decreased in order to comply with the electrostatic requirements. In principle, compensation of the surface polarity is achieved by the removal of 42% of the topmost O_3_ layer only. However, selectively removing this fraction only from the O_3_ part of the full O_3_–O evidently does not match the experimentally observed superstructure. Instead we do not discriminate between O in the O_3_–O layers, and therefore calculate that the total number of oxygen atoms in the O_3_–O oxygen layer has to be reduced from 4 to 2.74. Before we proceed to the model, it is relevant to first inspect the subsurface Al_3_ layer shown in Figure 3b, which is exposed when O is removed from the top layer. The underlying Al atoms constitute a so-called Kagomé lattice that, in this situation, may actually facilitate the creation of triangular shapes with a specific size and tentatively explain the experimentally observed structure. Considering that O needs to be removed in lines in order to comply with the observed structure, it is observed that Al has different densities along alternating lines on the Kagomé lattice, i.e., the density is a factor of two lower along the black line indicated in Figure 3b compared to the red line. Since undercoordinated Al is presumably associated with a high energy, we suggest that O is mainly removed along the low-density Al lines. Such circumstances clearly favor the formation of line vacancies, as compared to randomly distributed vacancies, which then, given the hexagonal surface crystal structure, drives the creation of triangular structures as observed in the experiments. Starting from a fully covered O layer, the removal of oxygen atoms along the thin black line in Figure 3b, leads to a factor of two fewer aluminium atoms exposed as compared to removal of oxygen atoms along the thin red line. Figure 3c illustrates a structural model of the best fit to the experimentally observed pattern, constructed from the considerations above. The model is constructed by removing triangles consisting of double O rows coordinated only to the low-density Al rows in the subsurface layers as shown in the ball model below Figure 3c. All other configurations do not match either the size or symmetry of the observed superstructure, or lead to significant deviations in the amount of O removed from the surface compared to the optimum. The model in Figure 3c reflects a very large 6**√**3×6**√**3R30° superstructure with a unit cell parameter of 6**√**3·*a*_hex_ = 5.9 nm, and the 30° orientation matches the experimentally observed structure. The amount of oxygen removed corresponds to 114 out of 432 per superstructure unit cell, leading to a total decrease in the initially O_4_ surface layer to O_2.9_, which is close to the theoretically predicted optimum amount of O_2.74_ required to stabilize the surface by charge removal alone. Any remaining surface polarity can be explained by the compensation originating from the step edges (Figure 2c) or scattered O vacancies, which are not imaged in the NC-AFM images. It is noted that the criteria used above do not unambiguously determine the observed structure, and the detailed features, such as the specific size of the triangular features, may be influenced by other factors such as vacancy repulsion and edge energies, which are not readily explained by our data. It should be possible to image the surface in atomic detail with NC-AFM, and such future studies may be able to clarify the role of additional O vacancies and also shed light on surface hydroxy groups (OH), which have been observed to play a role in the stabilization of the MgAl_2_O_4_(100) surface [12].

## Conclusion

The NC-AFM study presented in this work proposes a surface model for the polar MgAl_2_O_4_(111) surface. Under the assumption that the surface becomes terminated by an oxygen layer when synthesized in an oxygen atmosphere, a structural model is proposed that complies with both the electrostatic stabilization criterion for this polar surface, which requires the removal of ~42% of the surface oxygen, and fits with the size and triangular symmetry of the observed 6**√**3×6**√**3R30° superstructure. The preferred formation of the observed triangular shapes on an originally oxygen-terminated surface with hexagonal symmetry can be explained by the underlying Kagomé Al lattice, which is shown to facilitate the removal of oxygen in line vacancies with a triangular symmetry.

## Experimental

The experiments were performed in ultrahigh vacuum (UHV) with a base pressure better than 1 × 10^−10^ mbar. The UHV system is equipped with a combined STM/AFM microscope, X-ray photoelectron spectroscopy (XPS) and means for sample preparation. The MgAl_2_O_4_ single crystal used for this NC-AFM study was purchased from the MTI Corporation with an EPI polished (111) facet. The crystal was first rinsed in a 1:1 mixture of nitric acid (65%) and water followed by annealing in a furnace at a temperature of 1000 °C for 4 h. After introduction to the vacuum chamber the samples underwent cycles of Ar^+^ sputtering (5 min at 1 keV acceleration energy) followed by annealing (5 min at 1150 °C utilizing a 2 °C/s temperature up and down ramp) in a 1 × 10^−7^ mbar O_2_ atmosphere. After approximately 15 such cleaning cycles the crystal was sufficiently flat and clean for performing NC-AFM. We monitored the surface cleanliness by XPS using Mg Kα radiation (Phoibos 100 analyzer and XR 50 source, SPECS GmbH, Berlin, Germany). XPS spectra were recorded with the surface normal pointing in the direction of the analyzer and revealed the presence of only Mg, Al and O. XPS spectra were recorded regularly during the preparation, and the stoichiometry of the crystal was not observed to change as function of the number of preparation cycles.

For NC-AFM, silicon cantilevers from Nanoworld with a resonance frequency of 330 kHz and a force constant of 42 N/m were utilized. The constant-detuning mode was used for topographic imaging of the surface by fixing the detuning of the AFM cantilever at a specific setpoint (Δ*f*_set_) and recording the variation of the tip height (*z*) while raster scanning the surface. The surface potential, measured after annealing the crystal, was generally quite high, often in the range 4–8 V. Therefore, the voltage applied between the surface and the tip, *U*_bias_, was monitored and adjusted regularly to minimize the electrostatic forces arising from the contact potential difference.

## References

[1] Heinrich V E, Cox P A (1994). The Surface Science of Metal Oxides.

[2] Lauritsen J V, Reichling M (2010). J Phys: Condens Matter.

[3] Altman E I, Schwarz U D (2010). Adv Mater.

[4] Barth C, Foster A S, Henry C R, Shluger A L (2011). Adv Mater.

[5] Sickafus K E, Wills J M, Grimes N W (1999). J Am Ceram Soc.

[6] Tasker P W (1979). J Phys C: Solid State Phys.

[7] Noguera C (2000). J Phys: Condens Matter.

[8] Goniakowski J, Finocchi F, Noguera C (2008). Rep Prog Phys.

[9] Torbrügge S, Ostendorf F, Reichling M (2009). J Phys Chem C.

[10] Kresse G, Dulub O, Diebold U (2003). Phys Rev B.

[11] Lauritsen J V, Porsgaard S, Rasmussen M K, Jensen M C R, Bechstein R, Meinander K, Clausen B S, Helveg S, Wahl R, Kresse G (2011). ACS Nano.

[12] Rasmussen M K, Foster A S, Hinnemann B, Canova F F, Helveg S, Meinander K, Martin N M, Knudsen J, Vlad A, Lundgren E (2011). Phys Rev Lett.

[13] Vaz C A F, Prabhakaran D, Altman E I, Henrich V E (2009). Phys Rev B.

[14] Meyer W, Biedermann K, Gubo M, Hammer L, Heinz K (2008). J Phys: Condens Matter.

[15] Harding J H (1999). Surf Sci.

[16] Fang C M, Parker S C, de With G (2000). J Am Ceram Soc.

[17] Davies M J, Parker S C, Watson G W (1994). J Mater Chem.

[18] Fang C M, de With G, Parker S C (2001). J Am Ceram Soc.

[19] Fang C M, Parker S C, de With G (2001). Key Eng Mater.

[20] Méducin F, Redfern S A T, Le Godec Y, Stone H J, Tucker M G, Dove M T, Marshall W G (2004). Am Mineral.

[21] Ostendorf F, Torbrügge S, Reichling M (2008). Phys Rev B.

[22] Dulub O, Diebold U, Kresse G (2003). Phys Rev Lett.

[23] Sadewasser S, Lux-Steiner M C (2003). Phys Rev Lett.

